# A Deeper Union: From a Failed Project to the European Quality Lead

**DOI:** 10.1007/s10272-021-0975-8

**Published:** 2021-06-04

**Authors:** Karl Aiginger

**Affiliations:** c/o ÖGfE, Sekretariat Maxine Amstätter, Policy Crossover Center: Vienna - Europe, Rotenhausgasse 6, 1090 Vienna, Austria

## Abstract

After President Trump’s departure, many expected that the transatlantic partnership would return to its previous state with the US playing a leading role. This article challenges that view. Instead, a new world order is foreseen, with different partnerships and spheres of influence. Europe can decide whether it wants to remain small and homogeneous or a larger but also more heterogenous Union that leads in welfare indicators such as life expectancy, fighting poverty and limiting climate change. Expanding this lead and communicating its uniqueness can empower Europe to combine enlargement and deepening, which appears unlikely without changes in governance and self-confidence.

The COVID-19 crisis brought about new challenges and opportunities for the European Union, as it did for the entire world. After decades of eradication of extreme poverty and limiting inequality through taxes, transfers and cohesion policy, inequality between the periphery and the centre is again increasing and some regions feel forgotten. The population is ageing in many countries, and this is often exacerbated by the emigration of young people, making the regions they leave less attractive to new firms.

New technologies and digital transformation stimulate firm growth and could make life easier, but Europe lags in research and development (R&D) relative to the US and to the Asian tigers. Europe is still not attractive for the best brains due to the limited number of top universities.

The access to health services is easy, but coordination between member countries is limited, as demonstrated by emergency units and reserves in essential supplies. Testing procedures for new medications are accurate, which is extremely important for the quality of medicines and healthcare, but there should be a fast-track system in case of emergency, as has been painfully demonstrated by the current coronavirus pandemic.

Europe benefits from globalisation but is reluctant to fight to make it more socially and environmentally responsible so as to fit the European model. It has not set rules of responsibility for firms' suppliers (chain responsibility).

Additionally, the EU is lacking an immigration strategy. There is no blue card systems to make Europe attractive to top-qualified Indian or Chinese citizens. The EU does not seek to limit disruptive immigration through education and peace-building in Africa. Border countries take on a disproportionate burden of this disruptive immigration.

A strong position in the new world order requires a deeper Union to boost the EU's lead in sustainability, one that does not interfere but rather empowers and coordinates local solutions.

## Game changers on the road to a deeper Union

The following ten points of a reform agenda envisage a double strategy for deepening the Union as well as offering partnership with neighbours. This needs ambitious goals but empowering local innovations (Aiginger, 2017, 2021; Ketels and Porter, 2020).

### Dynamics

Economic growth is rather low in Europe. The EU failed at using information and communications technology to accelerate dynamics and was slow to recover from the financial crisis. During the COVID-19 crisis, the decline in

**Karl Aiginger**, Policy Crossover Center: Vienna – Europe, Vienna; University of Economics and Business Vienna, Austria.

GDP in the first year was stronger than in the US. This is also predicted for the combined loss and expected gain in 2021 and 2022, while China's economy is expected to expand in both years. The GDP per capita in the EU is one-third lower than in the US, and the catching-up process in labour productivity stopped in the last decades of the past century. Europe should not mimic the US model; income dynamics make it easier to prioritise equality and decarbonisation.

## Closing the research deficit

Modern growth theory tells us that insufficient research is a root cause of lacklustre growth. R&D expenditure as a share of GDP in the EU is about one-quarter lower than in the US. R&D spending should have been raised to 3% of GDP, according to the Lisbon 2000 strategy, which intended to make Europe the "most competitive region of the world". As this did not materialise, the EU 2020 programme tried to enforce a "national ownership of the strategy" by demanding that members set national targets. But, first, the sum of the national R&D goals did not amount to the EU target, and second, member countries failed to reach their own goals. Actual expenditure remained slightly above 2%, now trailing not only the US at 3%, but also the Asian tigers and, since 2012, China (OECD, 2014).

## Redirecting productivity

Productivity growth allows for the same output with less input. It is important to distinguish, however, which partial productivity increases: it can be labour productivity or energy and resource productivity.[1] These possibilities have different policy implications (for more details, see Aiginger, 2021).

Booming labour productivity exacerbates the growth imperative; if labour productivity grows by 3%, output must increase at the same rate. Otherwise, unemployment will increase and low-skilled workers will lose their jobs. Increasing energy and resource productivity also reduces inputs and stimulates cost competitiveness, while at the same time limiting emissions. Achieving the climate targets requires a reduction in greenhouse gases of 80% to 95%.

Redirecting productivity from its current focus does not represent an unjustified interference in a market economy. On the contrary, the current dominance of labour productivity follows from high taxes on labour and low taxes on energy and transport.

## Reducing inequality

Europe has to reduce inequality within countries, across regions, and – an aspect of increasing importance – between the core and the periphery (Aiginger and Kreuz, 2020), which would in turn limit nationalism. "Forgotten regions" are a powerful source of populism and illiberal democracy. This creates opposition to a deepening of the EU and its ability to cope with new challenges.

Traditional policy instruments are needed to limit inequality, since the bulk of taxes are levied on wages (where extremely high rates limit formal employment) or on consumption. This could take the form of a combination of standardising tax bases for corporate or inheritance taxes and pricing emissions, fighting base shifting and tax evasion. New taxes on financial speculations, plastic or platforms would also reduce inequality. To achieve this goal, member states must engage with each other, since taxes are a national matter.

## Reforming governance: Deepening and empowering

The request for unanimity of decisions in the European Council has to be removed. The agreement of a subgroup on an issue (enhanced cooperation) should be used more often and welcomed by all European institutions (today it depends on the pre-approval of the European Council).

The right of the European Parliament to start the legislative initiative should be established. For European elections, political parties at the European level should be given preference.

Member countries that defy European decisions and international compacts, or that do not respect human rights and waive the division of power between the executive and legislative power or limit the freedom of the media and universities should not receive substantial European grants and subsidies.

Reforms should target problems with common interests, strong external benefits and a high impact on future well-being. However, it is important to distinguish between principles and targets on the one hand and implementation on the other. Innovation is a process in which local initiatives reveal unexpected gains. Thus, a deeper Union that sets targets together and encourages bottom-up solutions is better than central planning, and also preferable to ambivalent, partly conflicting local priorities.

1 For the sake of simplicity, we put aside capital productivity (both human and physical capital).

To limit global warming, goals must be set and their implementation requires national and local initiatives. The central authority must request local plans, monitor their implementation and inform about best practices. It cannot be acceptable that some regions use renewable energy while other countries build new coal plants. Even cleaner heating with oil and gas is not compatible with climate neutrality in 2050. Massive investment in energy productivity and renewable energy, as well as better insulation of buildings, must occur in all regions.

A deeper Union in the sense of common goals is not the opposite of local initiatives; rather, it can encourage such initiatives and make them more effective. And a larger Union can gain the support of citizens, if they know that European leverage on a global scale depends on Europe negotiating together.

### From size to impact: Media and external communications

The visibility of Europe in international meetings and organisations should be heightened. A European seat in the UN, the WTO, the IMF or the WHO would first necessitate coordination between European countries, but then increase their impact on global decisions and the adherence of European countries to global decisions.

### From size to impact: Promoting the euro as a transaction currency

The EU should promote the use of the euro as a reserve currency alternative to the dominant dollar before the renminbi assumes this role. The share of transactions in euros is far lower than the trade volume of euro area members.[2] The creation of a euro debt market with high liquidity would provide for international investors looking for alternatives to the dollar, a process that began with the European Stability Mechanism. European safe assets should be promoted and the financing of the Recovery and Resilience Facility, with the same small common liability of all countries for new debt, is a small first step.

Currently only 23% of international loans are in euros (more than 50% are in dollars); the market share for the euro is at 16% of global foreign exchange turnover, and the share of the euro in global foreign exchange reserves is 20% (Baldwin and Weder di Mauro, 2020).

### From size to impact: Neighbourhood policy

A geopolitical role starts in the neighbourhood. Europe has to build partnerships with neighbours in the East and the South (Aiginger and Handler, 2018). Even if we argue that the share of Europe in GNP, exports and manufacturing is larger than perceived, it will shrink for a given geographical size. The EU can offer partnerships to its neighbours since it is a continent with soft power that does not intervene by military force. Europe's leading role in fighting climate change offers important technologies that "rising Africa" can adapt to local needs, such as digital payments and remote healthcare consultations, which have already been used successfully. European engagement in Africa is regarded with scepticism due to Europe's colonial past, and therefore the European Commission and members without former colonies will have to lead.

### The Recovery and Resilience Facility as a reform engine

The new Multiannual Financial Framework, as well as the Recovery and Resilience Facility (RRF), could boost reforms. The latter is partly financed by new European Safe Assets, with common guarantees. The European budget and the new facility should be used to increase dynamics (as measured by GDP), productivity and research, but even more support sustainability, energy and resource conservation, and new clean technologies. To achieve this, EU funding and national public expenditures have to coordinate their goals, given that the EU budget amounts to only 1% of GDP, while the national public expenditures amount to 40%. The additional expenditures via RRF increase the share of European expenditures for some years to 1.8%.

The RRF is a combination of mandatory national planning and supranational control. It demands a focus on reforms, decarbonisation and digital transformation. Member countries have to present regional plans in the first half of 2021 revealing national priorities. Fresh money cannot be used for old projects. Together with reforms in the European budget, this could redirect European policy.

### Changes have started

The new European Commission intends to reinforce Europe as a stronger partner at the global level and at the same time increase the commitment and responsibility to European citizens. The first has been stressed by Commissioner Ursula von der Leyen's intention to promote a "geopolitical Commission" and "a Europe closer to citizens, by supporting local led development strategies…. across the EU" (von der Leyen, 2019).

2 The euro has made inroads as a global payment currency, but its share of 40% is still five percentage points below that of the dollar, though Europe's trade share is nearly twice as high as that of the US (European Commission, 2018; European Central Bank, 2020).

## The new world will not be bipolar

The new world order will be different post-COVID-19. New challenges have come up gradually and have become more visible during the pandemic. Crises should make firms and governments rethink their position and investments. Changes that are not made today will be delayed for a long time. This year is a watershed moment.

Analysts predicting the future world order concur that it will be dominated by China and the US, but both countries have demonstrated their limits as of late. China is seen in a much more critical light due to domestic issues, but also because it extends its borders clandestinely and uses its investments to grab resources and create dependencies. The US plans to return to its former position, intent on assisting its firms so they can vanquish the competition, contrary to WTO rules. The US lags in most Sustainable Development Goals and in energy productivity. The Biden Administration cannot change this in the next two years since it may lose its majority in the midterm elections.

The power of the EU in world politics can only be achieved by deepening the Union. This starts by defining the topics that can best be solved together (and maybe those that can be returned to members). Climate change, digitalisation, ageing, migration and the health crisis all need to be and can be addressed more efficiently if the European governance system accelerates decisions, sets common goals and monitors adherence. The details need not be fixed centrally. On the contrary, due to new member countries, the heterogeneity of the EU is increasing and preferences become less homogeneous with rising incomes. The optimal strategy will therefore be to set goals centrally and with global perspectives, while operationalisation is organised in the member countries if not in the regions. This dual strategy will also help curtail populistic and nationalistic backlashes motivated by a neglect of regional diversity in the larger Union.

The autocratic tendencies in China and the political divide in the US should effectively encourage Europe to become more ambitious and self-confident. A deeper Union that at the same time encourages regional initiatives could allow Europe to take more responsibility for climate concerns, while limiting income differences and boosting cooperation with its buoyant neighbourhood. The result could be a multi-polar world, with Europe as a quality player that offers its model to its neighbours while learning from them.
